# GESAP trial rationale and methodology: management of patients with suspected obstructive sleep apnea in primary care units compared to sleep units 

**DOI:** 10.1038/s41533-016-0010-x

**Published:** 2017-02-07

**Authors:** Núria Tarraubella, Jordi de Batlle, Núria Nadal, Anabel L. Castro-Grattoni, Silvia Gómez, Manuel Sánchez-de-la-Torre, Ferran Barbé

**Affiliations:** 1 Group of Translational Research in Respiratory Medicine, Hospital Universitari Arnau de Vilanova and Santa Maria, IRBLleida, Rovira Roure, 80, Lleida, Spain; 2 Primary Care Unit of Tàrrega, Catalonia, Spain; 3 Direcció Atenció primària Àmbit Lleida, Catalonia, Spain; 4 Centro de Investigación Biomédica en Red de Enfermedades Respiratorias (CIBERES), Madrid, Spain

## Background

Obstructive sleep apnea syndrome (OSA) is a chronic sleep disorder characterized by repeated episodes of upper airway collapse during sleep. This leads to arterial hypoxemia and sleep disruption and causes daytime sleepiness and several associated dysfunctions, including cardiovascular, respiratory, metabolic, inflammatory, cognitive, and behavioral disorders.^1^ OSA is a relevant public health issue, with epidemiological studies showing a prevalence of 10% in middle-aged men and 3% in middle-aged women.^2^ Moreover, OSA has been associated with the development of cardiovascular events^3, 4^ and resistant hypertension,^5^ has a negative impact on quality of life,^6^ and has even been shown to have a causative role in traffic accidents.^7^ The application of continuous positive airway pressure (CPAP) is a highly effective treatment for OSA that can improve symptoms and quality of life, decrease traffic accidents and potentially lessen cardiovascular morbidity.^8, 9^ Furthermore, CPAP is cost-effective.^10^ However, only approximately 10% of individuals with OSA are diagnosed and treated. This scarcity in diagnosis has direct public health consequences due to the above-mentioned health implications and the high economic costs associated with untreated OSA.

Currently, the diagnosis and management of OSA are performed in highly specialized hospital-based sleep units (SUs), where full sleep studies (polysomnography (PSG)) or respiratory poligraphy (RP) can be conducted. However, this management approach has proven to be insufficient in identifying most OSA cases in the population, in addition to being cost-ineffective and generating long waiting lists.^11^ Given that OSA is a common chronic disorder, we believe that all levels of a healthcare system, especially primary care (PC), should be included in its management.^12–14^ The first trials assessing the management of OSA at the PC level reported satisfactory results.^15–19^ Moreover, our group showed that CPAP compliance did not differ between the PC and SU setting and was more cost-effective in the PC setting.^19^ However, in the above studies, although OSA management occurred at the PC level, diagnosis had always occurred in a SU. Therefore, in the current study, we aimed to determine the efficacy and cost-effectiveness of implementing a program for the diagnosis and management of OSA that can be conducted by PC personnel, and we compared these outcomes to those generated using the standard diagnosis and management protocols that are practiced in SUs.

### AIMS

The main objectives of the GESAP study are to assess the efficacies of PC and SU programs for OSA management. These assessments will be made using the Epworth sleepiness scale (ESS) before and for 6 months after initiating the program to assess its cost-effectiveness based on both ESS and quality of life (EuroQol-5D). Secondary objectives include assessments of patient satisfaction, treatment compliance, and the number, severity, and evolution of the treatment’s side effects.

## Methods

### Design

**Fig. 1:**
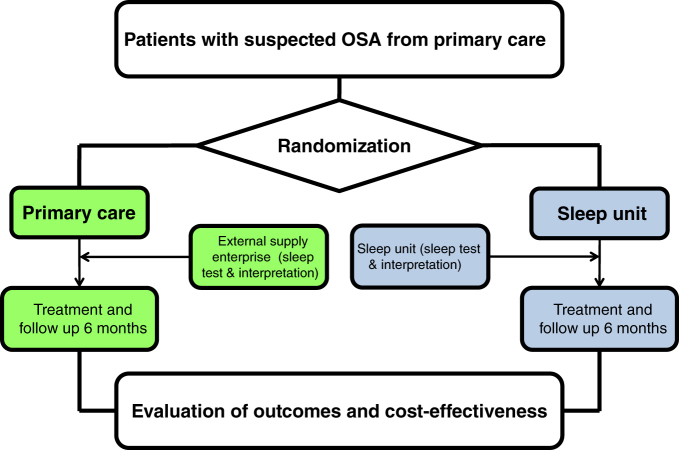
Study flow diagram

This is an open-label, parallel, prospective, randomized controlled trial. Figure 1 shows the study flow diagram. Patients will be consecutively included while attending visits with PC physicians due to suspected OSA or resistant hypertension. The included patients will be randomized by PC personnel to either PC or SU management in a 1:1 ratio. The patients randomized to SU management will be forwarded to the SU. Finally, after achieving an appropriate diagnosis and completing a 6-month course of follow-up, outcome evaluation and cost-effectiveness analysis will be performed. This research was approved by the Ethical Committee for Clinical Research of the Hospital Arnau de Vilanova—Santa Maria (Lleida, Spain), and the trial has been registered at ClinicalTrials.gov Id: NCT02234765.

### Subjects

The following inclusion criteria will be applied (Table 1): patients over 18 years of age visiting a PC unit in the region of Lleida, Spain, because of suspected OSA, based on the presence of chronic snoring, partner-objectified apnea and/or excessive daytime sleepiness or because of resistant hypertension (Alcarràs, Bordeta-Magraners, Borges Blanques, Cappont, Ciutat Jardí, Primer de Maig, El Pla d’Urgell, Ponts, Tàrrega, Rambla Ferran and, Balàfia-Pardinyes-Secà). All patients will be required to provide written informed consent.

**Table 1:** Inclusion/exclusion criteria

Inclusion criteria	Exclusion criteria
Men and women age >18 years	Pulmonary illness
Patient suspected to have OSA or resistant hypertension	Advanced heart failure (NYHA III or IV)
	Associated advanced pathology (including any active neoplasm or tumor)
	Psychiatric disorder
	Restless legs syndrome
	Pregnancy
	Another dyssomnia or parasomnia
	Previous treatment with CPAP

### Randomization and intervention

Randomization will be performed using an automated password-protected system, stratified by center. Each enrolled patient will be allocated in a 1:1 ratio to either the SU or PC arm. A total of 280 patients will be randomized (140:140). The patients randomized to the SU arm will be forwarded to the SU of Hospital Arnau de Vilanova—Santa Maria. In the SU, the patients will be diagnosed using PSG or RP as per usual clinical practice. Excessive daytime sleepiness will be measured using the validated Spanish version of the ESS.^20^ Patient follow-up and monitoring will be performed by SU personnel. In the PC arm, PC physicians will administer the ESS and request sleep testing from an external enterprise that supplies home respiratory therapies in the study area (the personnel of this enterprise have adequate training and expertise). Sleep tests will be performed using RP devices that meet the requirements for producing level III evidence proposed by the American Sleep Disorders Association. A full report will then be forwarded to the PC physician, who will choose an appropriate therapeutic option and perform monitoring and follow-up. The PC physicians will receive training from SU personnel before the study starts.

### CPAP treatment

CPAP treatment will be prescribed according to the following guidelines of the Spanish Society of Pneumology (SEPAR):^1^ an apnea-hypo apnea index (AHI) ≥30 h^−1^ regardless of the presence of cardiovascular comorbidity or hypersomnia or an AHI ≥ 5 h^−1^ accompanied by excessive daytime sleepiness (ESS ≥ 12) or cardiovascular comorbidity. CPAP titration will be performed using an automatic CPAP device (Autoset-T, Resmed, Sidney, Australia) according to previously described methodology.^21^

### Follow-up

All enrolled patients will undergo the same 6-month course of follow-up regardless of study arm. Patients undergoing CPAP treatment will be visited after 15 days, 1, 3, and 6 months to assess adherence to treatment (hours/day and pressure as provided by the CPAP device), solve potential issues related to CPAP utilization, and measure daytime sleepiness (ESS). CPAP pressure might be modified, when necessary, to better suit a patient’s requirements. Possible side effects of treatment as well as variables related to the care and maintenance of the CPAP equipment will be collected. Patients diagnosed with OSA who do not require CPAP treatment will be evaluated at 3 and 6 months. Finally, non-OSA patients will be visited only at 6 months. All follow-up visits will be held at the PC unit or SU according to the patient’s study arm.

### Study variables and data collection

The following variables will be collected at recruitment and at the 6-month follow-up: (i) clinical variables, including age, sex, daytime sleepiness (ESS), blood pressure, and drug use habits; (ii) anthropometric variables, including weight, height, BMI, and neck, waist and hip circumferences; (iii) comorbidities, including depression, anxiety, hypertension, heart, neurological, or respiratory diseases, non-active neoplasm, diabetes, and dyslipidemia; (iv) PSG or RP variables, including recording time, AHI, saturation <90% (CT90); and (v) quality of life (EuroQol-5D) and work or traffic accidents. Additionally, the compliance of patients undergoing CPAP treatment will be determined by dividing the number of hours of use (obtained from the internal clock of the CPAP device) by the number of days of treatment; adequate compliance will be defined as CPAP use ≥4 h/day. The final follow-up visit will also include an visual analog questionnaire on satisfaction about the received care in addition to an estimation of the overall direct costs of the treatment based on (i) sleep test costs (RP, PSG, and titration by automatic CPAP); (ii) CPAP treatment costs; (iii) patients’ travel costs; and (iv) healthcare costs 6 months before and after randomization, including number of PC and SU consultations and number and duration of emergency or day hospital admissions. Hospital and PC costs will be assessed using prices provided by the Catalan Institute of Health.^22^

### Sample size calculation

According to the literature, a calculation will be performed to support a non-inferiority analysis with ESS as the main study variable. A loss of 30% will be assumed,^23^ as well as an alpha error = 0.05, a beta error = 0.2, a non-inferiority limit of −2, and a common standard deviation for the two groups of ±5.9 for the principal variable.^24^

### Statistical analysis

Efficacy assessment will include per protocol analysis, excluding subjects who do not complete the follow-up. Adjusted analysis of covariance will be used to compare changes in ESS before and after the intervention in each study arm (non-inferiority margin = −2 points). *χ* ^2^ tests and *t*-tests (or non-parametric equivalents) will be used to compare quality of life (EuroQol-5D) and adherence to treatment in the 2 study arms as appropriate. Stratified analyses by CPAP treatment will be performed.

Cost-effectiveness assessment will include an intention-to-treat analysis to assess the total costs for each arm based on treatment effectiveness (ESS and EuroQol-5D). This will be performed using Bayesian cost-effectiveness techniques. The cost-effectiveness ratio of each treatment, the incremental cost-effectiveness ratio, the cost-effectiveness plane, the net benefit (NB) of each treatment, the incremental NB, and the cost-effectiveness acceptability curve will be considered.

## Discussion

We believe that satisfactory management of the diagnosis and treatment of patients with suspected OSA can be obtained in the PC setting by performing actions at various levels, including the interactive training of PC teams, the sharing of electronic medical records between PC units and SUs, and the use of current technology. We expect that the included patients will achieve a comparable level of clinical response, satisfaction, treatment compliance, and complication avoidance in both the PC and SU settings. In addition, we expect that PC management will be more cost-effective than the standard SU management. Overall, we believe that this project could establish a new paradigm for the management of a common condition that involves different levels of care as well as new technologies, and could demonstrate the ability to cost-effectively manage this chronic disease in the PC setting.
